# Status Report on Software Testing: Test-Comp 2021

**DOI:** 10.1007/978-3-030-71500-7_17

**Published:** 2021-02-24

**Authors:** Dirk Beyer

**Affiliations:** 8 Universidad Autónoma de Madrid, Madrid, Spain; 9 University of Twente, Enschede, The Netherlands; LMU Munich, Munich, Germany

**Keywords:** Software Testing, Test-Case Generation, Competition, Program Analysis, Software Validation, Software Bugs, Test Validation, Test-Comp, Benchmarking, Test Coverage, Bug Finding, Test-Suites, BenchExec, TestCov

## Abstract

This report describes Test-Comp 2021, the 3rd edition of the Competition on Software Testing. The competition is a series of annual comparative evaluations of fully automatic software test generators for C programs. The competition has a strong focus on reproducibility of its results and its main goal is to provide an overview of the current state of the art in the area of automatic test-generation. The competition was based on 3 173 test-generation tasks for C programs. Each test-generation task consisted of a program and a test specification (error coverage, branch coverage). Test-Comp 2021 had 11 participating test generators from 6 countries.

### 1 Introduction

Among several other objectives, the Competition on Software Testing (Test-Comp [4, 5, 6], https://test-comp.sosy-lab.org/2021) showcases every year the state of the art in the area of automatic software testing. This edition of Test-Comp is the 3rd edition of the competition. It provides an overview of the currently achieved results by tool implementations that are based on the most recent ideas, concepts, and algorithms for fully automatic test generation. This competition report describes the (updated) rules and definitions, presents the competition results, and discusses some interesting facts about the execution of the competition experiments. The setup of Test-Comp is similar to SV-COMP [8], in terms of both technical and procedural organization. The results are collected via BenchExec's XML results format [16], and transformed into tables and plots in several formats (https://test-comp.sosy-lab.org/2021/results/). All results are available in artifacts at Zenodo (Table 3).

This report extends previous reports on Test-Comp [4, 5, 6].
Reproduction packages are available on Zenodo (see Table 3).
Funded in part by the Deutsche Forschungsgemeinschaft (DFG) – 418257054 (Coop).
dirk.beyer@sosy-lab.org

**Competition Goals.** In summary, the goals of Test-Comp are the following [5]:

- Establish *standards* for software test generation. This means, most prominently, to develop a standard for marking input values in programs, define an exchange format for test suites, agree on a specification language for test-coverage criteria, and define how to validate the resulting test suites.
- Establish a set of *benchmarks* for software testing in the community. This means to create and maintain a set of programs together with coverage criteria, and to make those publicly available for researchers to be used in performance comparisons when evaluating a new technique.
- Provide an overview of *available tools* for test-case generation and a snapshot of the state-of-the-art in software testing to the community. This means to compare, independently from particular paper projects and specific techniques, different test generators in terms of effectiveness and performance.
- Increase the visibility and credits that *tool developers* receive. This means to provide a forum for presentation of tools and discussion of the latest technologies, and to give the participants the opportunity to publish about the development work that they have done.
- Educate PhD students and other participants on how to set up performance experiments, package tools in a way that supports reproduction, and how to perform *robust and accurate research experiments*.
- Provide *resources* to development teams that do not have sufficient computing resources and give them the opportunity to obtain results from experiments on large benchmark sets.

**Related Competitions.** In the field of formal methods, competitions are respected as an important evaluation method and there are many competitions [2]. We refer to the previous report [5] for a more detailed discussion and give here only the references to the most related competitions [2, 8, 32, 39].

**Quick Summary of Changes.** As the competition continuously improves, we report the changes since the last report. We list a summary of five new items in Test-Comp 2021 as overview:

- Extended task-definition format, version 2.0: Sect. 2
- SPDX identification of licenses in SV-Benchmarks collection: Sect. 2
- Extension of the SV-Benchmarks collection by several categories: Sect. 3
- Elimination of competition-specific functions `__VERIFIER_error` and `__VERIFIER_assume` from the test-generation tasks (and rules): Sect. 3
- CoVeriTeam: New tool that can be used to remotely execute test-generation runs on the competition machines: Sect. 4

### 2 Definitions, Formats, and Rules

Organizational aspects such as the classification (automatic, off-site, reproducible, jury, training) and the competition schedule is given in the initial competition definition [4]. In the following, we repeat some important definitions that are necessary to understand the results.

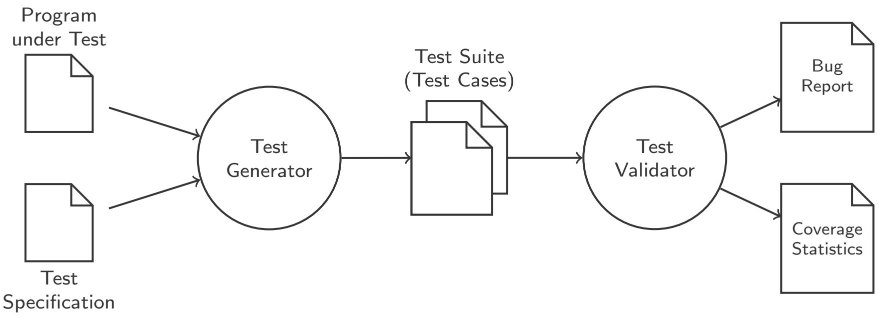

Fig. 1: Flow of the Test-Comp execution for one test generator (taken from [5])

**Test-Generation Task.** A *test-generation task* is a pair of an input program (program under test) and a test specification. A *test-generation run* is a non-interactive execution of a test generator on a single test-generation task, in order to generate a test suite according to the test specification. A *test suite* is a sequence of test cases, given as a directory of files according to the format for exchangeable test-suites.[1]

**Execution of a Test Generator.** Figure 1 illustrates the process of executing one test generator on the benchmark suite. One test run for a test generator gets as input (i) a program from the benchmark suite and (ii) a test specification (cover bug, or cover branches), and returns as output a test suite (i.e., a set of test cases). The test generator is contributed by a competition participant as a software archive in ZIP format. The test runs are executed centrally by the competition organizer. The test-suite validator takes as input the test suite from the test generator and validates it by executing the program on all test cases: for bug finding it checks if the bug is exposed and for coverage it reports the coverage. We use the tool TestCov [15][2] as test-suite validator.

**Test Specification.** The specification for testing a program is given to the test generator as input file (either `properties/coverage-error-call.prp` or `properties/coverage-branches.prp` for Test-Comp 2021).

The definition `init(main())` is used to define the initial states of the program under test by a call of function `main` (with no parameters). The definition `FQL(f)` specifies that coverage definition `f` should be achieved. The FQL (FShell query language [28]) coverage definition `COVER EDGES(@DECISIONEDGE)` means that all branches should be covered (typically used to obtain a standard test suite for quality assurance) and `COVER EDGES(@CALL(foo))` means that a call (at least one) to function `foo` should be covered (typically used for bug finding). A complete specification looks as follows: `COVER( init(main()), FQL(COVER EDGES(@DECISIONEDGE)) )`.

[1] https://gitlab.com/sosy-lab/software/test-format/
[2] https://gitlab.com/sosy-lab/software/test-suite-validator

Table 1: Coverage specifications used in Test-Comp 2021 (similar to 2019, 2020)

| Formula | Interpretation |
| --- | --- |
| `COVER EDGES(@CALL(reach_error))` | The test suite contains at least one test that executes function `reach_error`. |
| `COVER EDGES(@DECISIONEDGE)` | The test suite contains tests such that all branches of the program are executed. |

```
format_version: '2.0'

## old file name: floppy_true-unreach-call_true-valid-memsafety.i.cil.c
input_files: 'floppy.i.cil-3.c'

properties:
  - property_file: ../properties/unreach-call.prp
    expected_verdict: true
  - property_file: ../properties/valid-memsafety.prp
    expected_verdict: false
    subproperty: valid-memtrack
  - property_file: ../properties/coverage-branches.prp options:
  language: C
  data_model: ILP32
```

Fig. 2: Example task definition file `floppy.i.cil-3.yml` for C program `floppy.i.cil-3.c` (format version and options are new compared to last year)

Table 1 lists the two FQL formulas that are used in test specifications of Test-Comp 2021; there was no change from 2020 (except that special function `__VERIFIER_error` does not exist anymore).

**Task-Definition Format 2.0.** The format for the task definitions in the SV-Benchmarks repository was extended by options that can carry information from the test-generation task to the test tool. Test-Comp 2021 used the format in version 2.0 (`https://gitlab.com/sosy-lab/benchmarking/task-definition-format/-/tree/2.0`). The options now contain the language (C or Java) and the data model (ILP32, LP64, see `http://www.unix.org/whitepapers/64bit.html`, only for C programs) that the program of the test-generation task assumes (`https://github.com/sosy-lab/sv-benchmarks#task-definitions`). An example task definition is provided in Fig. 2: This YAML file specifies, for the C program `floppy.i.cil-3.c`, two verification tasks (reachability of a function call and memory safety) and one test-generation task (coverage of all branches). Previously, the options for language and data model where defined in category-specific configuration files (for example c/ReachSafety-ControlFlow.cfg), which were deleted before Test-Comp 2021.

**License and Qualification.** The license of each participating test generator must allow its free use for reproduction of the competition results. Details on qualification criteria can be found in the competition report of Test-Comp 2019 [6]. Furthermore, the community tries to apply the SPDX standard (`https://spdx.dev`) to the SV-Benchmarks repository. Continuous-integration checks based on REUSE (`https://reuse.software`) will ensure that all benchmark tasks adhere to the standard.

### 3 Categories and Scoring Schema

**Benchmark Programs.** The input programs were taken from the largest and most diverse open-source repository of software-verification and test-generation tasks[3], which is also used by SV-COMP [8]. As in 2020, we selected all programs for which the following properties were satisfied (see issue on GitHub[4] and report [6]):

1. compiles with `gcc`, if a harness for the special methods[5] is provided,
2. should contain at least one call to a nondeterministic function,
3. does not rely on nondeterministic pointers,
4. does not have expected result 'false' for property 'termination', and
5. has expected result 'false' for property 'unreach-call' (only for category *Error Coverage*).

This selection yielded a total of 3 173 test-generation tasks, namely 607 tasks for category *Error Coverage* and 2 566 tasks for category *Code Coverage*. The test-generation tasks are partitioned into categories, which are listed in Tables 6 and 7 and described in detail on the competition web site.[6] Figure 3 illustrates the category composition.

The programs in the benchmark collection contained functions `__VERIFIER_error` and `__VERIFIER_assume` that had a specific predefined meaning. Last year, those functions were removed from all programs in the SV-Benchmarks collection. More about the reasoning is explained in the SV-COMP 2021 competition report [8].

**Category Error-Coverage.** The first category is to show the abilities to discover bugs. The benchmark set consists of programs that contain a bug. Every run will be started by a batch script, which produces for every tool and every test-generation task one of the following scores: 1 point, if the validator succeeds in executing the program under test on a generated test case that explores the bug (i.e., the specified function was called), and 0 points, otherwise.

[3] `https://github.com/sosy-lab/sv-benchmarks`
[4] `https://github.com/sosy-lab/sv-benchmarks/pull/774`
[5] `https://test-comp.sosy-lab.org/2021/rules.php`
[6] `https://test-comp.sosy-lab.org/2021/benchmarks.php`

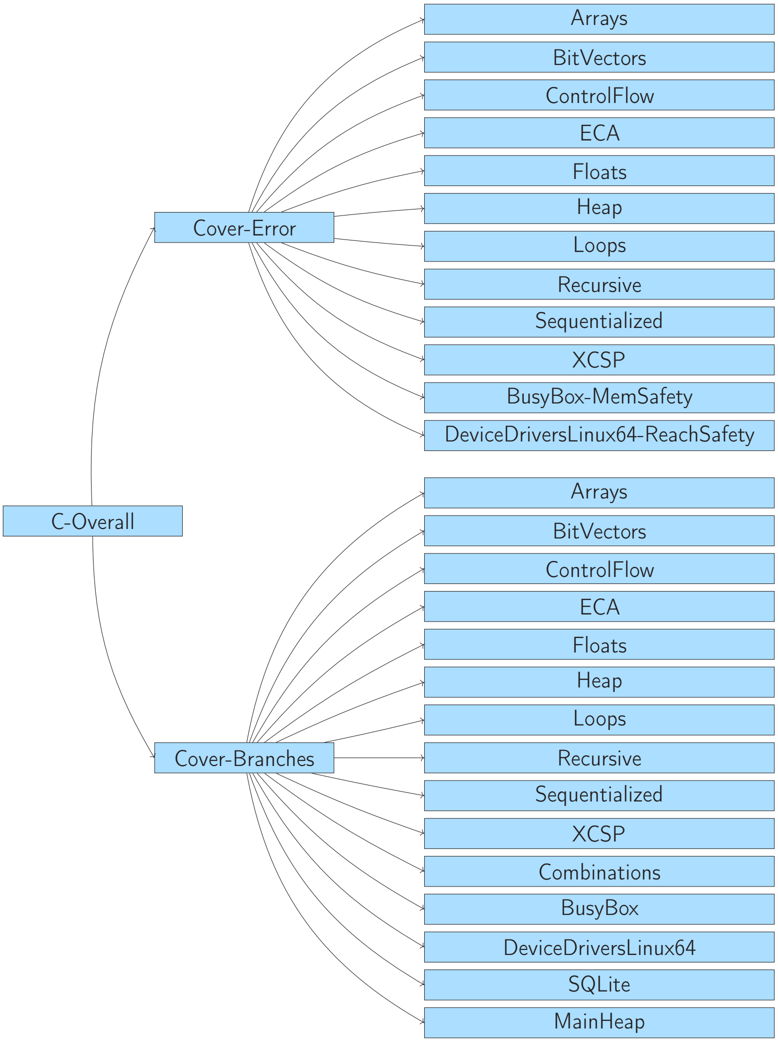

Fig. 3: Category structure for Test-Comp 2021; compared to Test-Comp 2020, there are three new sub-categories in *Cover-Error* and two new sub-categories in *Cover-Branches*: we added the sub-categories *XCSP*, *BusyBox-MemSafety*, and *DeviceDriversLinux64-ReachSafety* to category *Cover-Error*, and the sub-categories *XCSP* and *Combinations* to category *Cover-Branches*

**Category Branch-Coverage.** The second category is to cover as many branches of the program as possible. The coverage criterion was chosen because many test generators support this standard criterion by default. Other coverage criteria can be reduced to branch coverage by transformation [27]. Every run will be started by a batch script, which produces for every tool and every test-generation task the coverage of branches of the program (as reported by TestCov [15]; a value between 0 and 1) that are executed for the generated test cases. The score is the returned coverage.

**Ranking.** The ranking was decided based on the sum of points (normalized for meta categories). In case of a tie, the ranking was decided based on the run time, which is the total CPU time over all test-generation tasks. Opt-out from categories was possible and scores for categories were normalized based on the number of tasks per category (see competition report of SV-COMP 2013 [3], page 597).

### 4 Reproducibility

In order to support independent reproduction of the Test-Comp results, we made all major components that are used for the competition available in public version-control repositories. An overview of the components that contribute to the reproducible setup of Test-Comp is provided in Fig. 4, and the details are given in Table 2. We refer to the report of Test-Comp 2019 [6] for a thorough description of all components of the Test-Comp organization and how we ensure that all parts are publicly available for maximal reproducibility.

In order to guarantee long-term availability and immutability of the test-generation tasks, the produced competition results, and the produced test suites, we also packaged the material and published it at Zenodo (see Table 3). The archive for the competition results includes the raw results in BenchExec's XML exchange format, the log output of the test generators and validator, and a mapping from file names to SHA-256 hashes. The hashes of the files are useful for validating the exact contents of a file, and accessing the files inside the archive that contains the test suites.

To provide transparent access to the exact versions of the test generators that were used in the competition, all test-generator archives are stored in a public Git repository. GitLab was used to host the repository for the test-generator archives due to its generous repository size limit of 10 GB.

**Competition Workflow.** As illustrated in Fig. 4, the ingredients for a test or verification run are (a) a test or verification task (which program and which specification to use), (b) a benchmark definition (which categories and which options to use), (c) a tool-info module (uniform way to access a tool's version string and the command line to invoke), and (d) an archive that contains all executables that are required and cannot be installed as standard Ubuntu package.

(a) Each test or verification task is defined by a task-definition file (as shown, e.g., in Fig. 2). The tasks are stored in the SV-Benchmarks repository and maintained by the verification and testing community, including the competition participants and the competition organizer.

(b) A benchmark definition defines the choices of the participating team, that is, which categories to execute the test generator on and which parameters to pass to the test generator. The benchmark definition also specifies the resource limits of the competition runs (CPU time, memory, CPU cores). The benchmark definitions are created or maintained by the teams and the organizer.

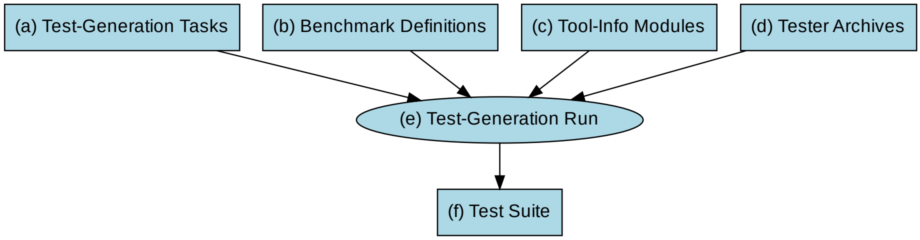

Fig. 4: Benchmarking components of Test-Comp and competition's execution flow (same as for Test-Comp 2020)

Table 2: Publicly available components for reproducing Test-Comp 2021

| Component | Fig. 4 | Repository | Version |
|---|---|---|---|
| Test-Generation Tasks | (a) | github.com/sosy-lab/sv-benchmarks | testcomp21 |
| Benchmark Definitions | (b) | gitlab.com/sosy-lab/test-comp/bench-defs | testcomp21 |
| Tool-Info Modules | (c) | github.com/sosy-lab/benchexec | 3.6 |
| Test-Generator Archives | (d) | gitlab.com/sosy-lab/test-comp/archives-2021 | testcomp21 |
| Benchmarking | (e) | github.com/sosy-lab/benchexec | 3.6 |
| Test-Suite Format | (f) | gitlab.com/sosy-lab/software/test-format | testcomp21 |

Table 3: Artifacts published for Test-Comp 2021

| Content | DOI | Reference |
|---|---|---|
| Test-Generation Tasks | 10.5281/zenodo.4459132 | [9] |
| Competition Results | 10.5281/zenodo.4459470 | [7] |
| Test Suites (Witnesses) | 10.5281/zenodo.4459466 | [10] |
| BenchExec | 10.5281/zenodo.4317433 | [43] |

(c) A tool-info module is a component that provides a uniform way to access the test-generation or verification tool: it provides interfaces for accessing the version string of a test generator and assembles the command-line from the information given in the benchmark definition and task definition. The tool-info modules are written by the participating teams with the help of the BenchExec maintainer and others.

(d) A test generator is provided as an archive in ZIP format. The archive contains a directory with a README and LICENSE file as well as all components that are necessary for the test generator to be executed. This archive is created by the participating team and merged into the central repository via a merge request.

All above components are reviewed by the competition jury and improved according to the comments from the reviewers by the teams and the organizer.

Table 4: Competition candidates with tool references and representing jury members

| Tester | Ref. | Jury member | Affiliation |
|---|---|---|---|
| CMA-ES Fuzz | [33] | Gidon Ernst | LMU Munich, Germany |
| CoVeriTest | [12, 31] | Marie-Christine Jakobs | TU Darmstadt, Germany |
| FuSeBMC | [1, 25] | Kaled Alshmrany | U. of Manchester, UK |
| HybridTiger | [18, 38] | Sebastian Ruland | TU Darmstadt, Germany |
| Klee | [19, 20] | Martin Nowack | Imperial College London, UK |
| Legion | [37] | Dongge Liu | U. of Melbourne, Australia |
| LibKluzzer | [35] | Hoang M. Le | U. of Bremen, Germany |
| PRTest | [14, 36] | Thomas Lemberger | LMU Munich, Germany |
| Symbiotic | [21, 22] | Marek Chalupa | Masaryk U., Brno, Czechia |
| TracerX | [29, 30] | Joxan Jaffar | National U. of Singapore, Singapore |
| VeriFuzz | [23] | Raveendra Kumar M. | Tata Consultancy Services, India |

Due to the reproducibility requirements and high level of automation that is necessary for a competition like Test-Comp, participating in the competition is also a challenge itself: package the tool, provide meaningful log output, specify the benchmark definition, implement a tool-info module, and troubleshoot in case of problems. Test-Comp is a friendly and helpful community, and problems are reported in a GitLab issue tracker, where the organizer and the other teams help fixing the problems.

To provide participants access to the actual competition machines, the competition used CoVeriTeam [13] (`https://gitlab.com/sosy-lab/software/coveriteam/`) for the first time. CoVeriTeam is a tool for cooperative verification, which enables remote execution of test-generation or verification runs directly on the competition machines (among its many other features). This possibility was found to be a valuable service for trouble shooting.

### 5 Results and Discussion

For the third time, the competition experiments represent the state of the art in fully automatic test generation for whole C programs. The report helps in understanding the improvements compared to last year, in terms of effectiveness (test coverage, as accumulated in the score) and efficiency (resource consumption in terms of CPU time). All results mentioned in this article were inspected and approved by the participants.

**Participating Test Generators.** Table 4 provides an overview of the participating test generators and references to publications, as well as the team representatives of the jury of Test-Comp 2021. (The competition jury consists of the chair and one member of each participating team.) Table 5 lists the features and technologies that are used in the test generators. An online table with information about all participating systems is provided on the competition web site.[7]

[7] `https://test-comp.sosy-lab.org/2021/systems.php`

Table 5: Technologies and features that the competition candidates used

| Participant | Bounded Model Checking | CEGAR | Evolutionary Algorithms | Explicit-Value Analysis | Floating-Point Arithmetics | Guidance by Coverage Measures | Predicate Abstraction | Random Execution | Symbolic Execution | Targeted Input Generation | Algorithm Selection | Portfolio |
|---|---|---|---|---|---|---|---|---|---|---|---|---|
| CMA-ES Fuzz | | | ✓ | ✓ | | ✓ | | ✓ | | | | |
| CoVeriTest | | ✓ | | ✓ | ✓ | | ✓ | | | | | ✓ |
| FuSeBMC | ✓ | | | | ✓ | ✓ | | | | ✓ | | ✓ |
| HybridTiger | | ✓ | | ✓ | ✓ | | ✓ | | | | | |
| Klee | | | | | ✓ | | | | ✓ | ✓ | | |
| Legion | | | | ✓ | | ✓ | | ✓ | ✓ | ✓ | | |
| LibKluzzer | | | | | | ✓ | | ✓ | ✓ | | | |
| PRTest | | | | | | | | ✓ | | | | |
| Symbiotic | | | | | | ✓ | | | ✓ | ✓ | | ✓ |
| TracerX | ✓ | | | | | | | | ✓ | ✓ | | |
| VeriFuzz | ✓ | | ✓ | ✓ | | ✓ | | ✓ | | | | |

**Computing Resources.** The computing environment and the resource limits were the same as for Test-Comp 2020 [5]: Each test run was limited to 8 processing units (cores), 15 GB of memory, and 15 min of CPU time. The test-suite validation was limited to 2 processing units, 7 GB of memory, and 5 min of CPU time. The machines for running the experiments are part of a compute cluster that consists of 168 machines; each test-generation run was executed on an otherwise completely unloaded, dedicated machine, in order to achieve precise measurements. Each machine had one Intel Xeon E3-1230 v5 CPU, with 8 processing units each, a frequency of 3.4 GHz, 33 GB of RAM, and a GNU/Linux operating system (x86_64-linux, Ubuntu 20.04 with Linux kernel 5.4). We used BenchExec [16] to measure and control computing resources (CPU time, memory, CPU energy) and VerifierCloud [8] to distribute, install, run, and clean-up test-case generation runs, and to collect the results. The values

[8] https://vcloud.sosy-lab.org

Table 6: Quantitative overview over all results; empty cells mark opt-outs; label 'new' indicates first-time participants

| Participant | **Cover-Error** 607 tasks | **Cover-Branches** 2566 tasks | **Overall** 3173 tasks |
|---|---|---|---|
| CMA-ES Fuzz new | 0 | 411 | 254 |
| CoVeriTest | 225 | 1128 | 1286 |
| FuSeBMC new | **405** | 1161 | **1776** |
| HybridTiger | 266 | 860 | 1228 |
| Klee | 339 | 784 | 1370 |
| Legion | 35 | 651 | 495 |
| LibKluzzer | **359** | **1292** | **1738** |
| PRTest | 79 | 519 | 526 |
| Symbiotic | 314 | **1169** | 1543 |
| TracerX | 246 | 1087 | 1315 |
| VeriFuzz | **385** | **1389** | **1865** |

for time and energy are accumulated over all cores of the CPU. To measure the CPU energy, we use CPU Energy Meter [17] (integrated in BenchExec [16]). Further technical parameters of the competition machines are available in the repository which also contains the benchmark definitions. [9]

One complete test-generation execution of the competition consisted of 34 903 single test-generation runs. The total CPU time was 220 days and the consumed energy 56 kWh for one complete competition run for test generation (without validation). Test-suite validation consisted of 34 903 single test-suite validation runs. The total consumed CPU time was 6.3 days. Each tool was executed several times, in order to make sure no installation issues occur during the execution. Including preruns, the infrastructure managed a total of 210 632 test-generation runs (consuming 1.8 years of CPU time) and 207 459 test-suite validation runs (consuming 27 days of CPU time). We did not measure the CPU energy during preruns.

**Quantitative Results.** Table 6 presents the quantitative overview of all tools and all categories. The head row mentions the category and the number of test-generation tasks in that category. The tools are listed in alphabetical order; every table row lists the scores of one test generator. We indicate the top three candidates by formatting their scores in bold face and in larger font size. An empty table cell means that the test generator opted-out from the respective main category

[9] https://gitlab.com/sosy-lab/test-comp/bench-defs/tree/testcomp21

Table 7: Overview of the top-three test generators for each category (measurement values for CPU time and energy rounded to two significant digits)

| Rank | Tester | Score | CPU Time (in h) | CPU Energy (in kWh) |
|---|---|---|---|---|
| ***Cover-Error*** | | | | |
| 1 | **FuSeBMC** | **405** | 22 | 0.26 |
| 2 | VeriFuzz | 385 | 2.6 | 0.031 |
| 3 | LibKluzzer | 359 | 90 | 0.99 |
| ***Cover-Branches*** | | | | |
| 1 | **VeriFuzz** | **1389** | 630 | 8.1 |
| 2 | LibKluzzer | 1292 | 520 | 5.7 |
| 3 | Symbiotic | 1169 | 440 | 5.1 |
| ***Overall*** | | | | |
| 1 | **VeriFuzz** | **1865** | 640 | 8.1 |
| 2 | FuSeBMC | 1776 | 410 | 4.8 |
| 3 | LibKluzzer | 1738 | 610 | 6.7 |

(perhaps participating in subcategories only, restricting the evaluation to a specific topic). More information (including interactive tables, quantile plots for every category, and also the raw data in XML format) is available on the competition web site [10] and in the results artifact (see Table 3). Table 7 reports the top three test generators for each category. The consumed run time (column 'CPU Time') is given in hours and the consumed energy (column 'Energy') is given in kWh.

**Score-Based Quantile Functions for Quality Assessment.** We use score-based quantile functions [16] because these visualizations make it easier to understand the results of the comparative evaluation. The web site [10] and the results artifact (Table 3) include such a plot for each category; as example, we show the plot for category *Overall* (all test-generation tasks) in Fig. 5. All 11 test generators participated in category *Overall*, for which the quantile plot shows the overall performance over all categories (scores for meta categories are normalized [3]). A more detailed discussion of score-based quantile plots for testing is provided in the previous competition report [6].

**Alternative Rankings.** Table 8 is similar to Table 7, but contains the alternative ranking categories *Green Testing* and *New Test Generators*. Column 'Quality' gives the score in score points (sp), column 'CPU Time' the CPU usage in hours (h), column 'CPU Energy' the CPU usage in kilo-watt-hours (kWh), and column 'Rank Measure' reports the values for the rank measure, which is different for the two alternative ranking categories. (An entry '–' for 'CPU Energy' indicates that we did not measure the energy consumption for technical reasons.)

[10] `https://test-comp.sosy-lab.org/2021/results`

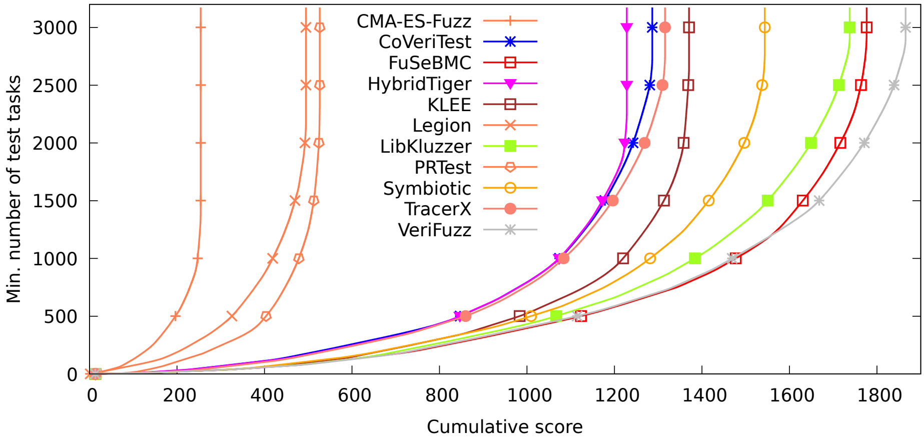

Fig. 5: Quantile functions for category *Overall*. Each quantile function illustrates the quantile ($x$-coordinate) of the scores obtained by test-generation runs below a certain number of test-generation tasks ($y$-coordinate). More details were given previously [6]. The graphs are decorated with symbols to make them better distinguishable without color.

Table 8: Alternative rankings; quality is given in score points (sp), CPU time in hours (h), energy in kilo-watt-hours (kWh), the first rank measure in kilo-joule per score point (kJ/sp), and the second rank measure in score points (sp); measurement values are rounded to 2 significant digits

| Rank | Test Generator | Quality (sp) | CPU Time (h) | CPU Energy (kWh) | Rank Measure |
|---|---|---|---|---|---|
| ***Green Testing*** | | | | | **(kJ/sp)** |
| 1 | **TRACERX** | 1 315 | 210 | 2.5 | 6.8 |
| 2 | KLEE | 1 370 | 210 | 2.6 | 6.8 |
| 3 | FUSEBMC | 1 776 | 410 | 4.8 | 9.7 |
| worst | | | | | 51 |
| ***New Test Generators*** | | | | | **(sp)** |
| 1 | **FUSEBMC** | 1 776 | 410 | 4.8 | 1 776 |
| 2 | CMA-ES FUZZ | 254 | 310 | – | 254 |

*Green Testing — Low Energy Consumption.* Since a large part of the cost of test generation is caused by the energy consumption, it might be important to also consider the energy efficiency in rankings, as complement to the official Test-Comp ranking. This alternative ranking category uses the energy consumption per score point as rank measure: $\frac{\text{CPU Energy}}{\text{Quality}}$, with the unit kilo-joule per

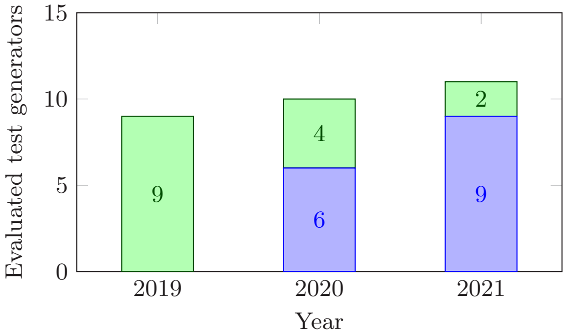

Fig. 6: Number of evaluated test generators for each year (top: number of first-time participants; bottom: previous year's participants)

score point ($kJ/sp$).[11] The energy is measured using CPU Energy Meter [17], which we use as part of BenchExec [16].

*New Test Generators.* To acknowledge the test generators that participated for the first time in Test-Comp, the second alternative ranking category lists measures only for the new test generators, and the rank measure is the quality with the unit score point (sp). For example, CMA-ES Fuzz is an early prototype and has already obtained a total score of 411 points in category *Cover-Branches*, and FuSeBMC is a new tool based on some mature components and became second place already in its first participation. This should encourage developers of test generators to participate with new tools of any maturity level.

### 6 Conclusion

Test-Comp 2021 was the the 3rd edition of the Competition on Software Testing, and attracted 11 participating teams (see Fig. 6 for the participation numbers and Table 4 for the details). The competition offers an overview of the state of the art in automatic software testing for C programs. The competition does not only execute the test generators and collect results, but also validates the achieved coverage of the test suites, based on the latest version of the test-suite validator TestCov. As before, the jury and the organizer made sure that the competition follows the high quality standards of the FASE conference, in particular with respect to the important principles of fairness, community support, and transparency.

**Data Availability Statement.** The test-generation tasks and results of the competition are published at Zenodo, as described in Table 3. All components and data that are necessary for reproducing the competition are available in public version repositories, as specified in Table 2. Furthermore, the results are presented online on the competition web site for easy access: `https://test-comp.sosy-lab.org/2021/results/`.

[11] Errata: Table 8 of last year's report for Test-Comp 2020 contains a typo: The unit of the energy consumption per score point is kJ/sp (instead of J/sp).

## References

[1] Alshmrany, K., Menezes, R., Gadelha, M., Cordeiro, L.: FuSeBMC: A white-box fuzzer for finding security vulnerabilities in C programs (competition contribution). In: Proc. FASE. LNCS 12649, Springer (2021)

[2] Bartocci, E., Beyer, D., Black, P.E., Fedyukovich, G., Garavel, H., Hartmanns, A., Huisman, M., Kordon, F., Nagele, J., Sighireanu, M., Steffen, B., Suda, M., Sutcliffe, G., Weber, T., Yamada, A.: TOOLympics 2019: An overview of competitions in formal methods. In: Proc. TACAS (3). pp. 3–24. LNCS 11429, Springer (2019). 10.1007/978-3-030-17502-3_1

[3] Beyer, D.: Second competition on software verification (Summary of SV-COMP 2013). In: Proc. TACAS. pp. 594–609. LNCS 7795, Springer (2013). 10.1007/978-3-642-36742-7_43

[4] Beyer, D.: Competition on software testing (Test-Comp). In: Proc. TACAS (3). pp. 167–175. LNCS 11429, Springer (2019). 10.1007/978-3-030-17502-3_11

[5] Beyer, D.: Second competition on software testing: Test-Comp 2020. In: Proc. FASE. pp. 505–519. LNCS 12076, Springer (2020). 10.1007/978-3-030-45234-6_25

[6] Beyer, D.: First international competition on software testing (Test-Comp 2019). Int. J. Softw. Tools Technol. Transf. (2021)

[7] Beyer, D.: Results of the 3rd Intl. Competition on Software Testing (Test-Comp 2021). Zenodo (2021). 10.5281/zenodo.4459470

[8] Beyer, D.: Software verification: 10th comparative evaluation (SV-COMP 2021). In: Proc. TACAS (2). LNCS 12652, Springer (2021), preprint available

[9] Beyer, D.: SV-Benchmarks: Benchmark set of 3rd Intl. Competition on Software Testing (Test-Comp 2021). Zenodo (2021). 10.5281/zenodo.4459132

[10] Beyer, D.: Test suites from Test-Comp 2021 test-generation tools. Zenodo (2021). 10.5281/zenodo.4459466

[11] Beyer, D., Chlipala, A.J., Henzinger, T.A., Jhala, R., Majumdar, R.: Generating tests from counterexamples. In: Proc. ICSE. pp. 326–335. IEEE (2004). 10.1109/ICSE.2004.1317455

[12] Beyer, D., Jakobs, M.C.: CoVeriTest: Cooperative verifier-based testing. In: Proc. FASE. pp. 389–408. LNCS 11424, Springer (2019). 10.1007/978-3-030-16722-6_23

[13] Beyer, D., Kanav, S.: CoVeriTeam: On-demand composition of cooperative verification systems. unpublished manuscript (2021)

[14] Beyer, D., Lemberger, T.: Software verification: Testing vs. model checking. In: Proc. HVC. pp. 99–114. LNCS 10629, Springer (2017). 10.1007/978-3-319-70389-3_7

[15] Beyer, D., Lemberger, T.: TestCov: Robust test-suite execution and coverage measurement. In: Proc. ASE. pp. 1074–1077. IEEE (2019). 10.1109/ASE.2019.00105

[16] Beyer, D., Löwe, S., Wendler, P.: Reliable benchmarking: Requirements and solutions. Int. J. Softw. Tools Technol. Transfer **21**(1), 1–29 (2019). 10.1007/s10009-017-0469-y

[17] Beyer, D., Wendler, P.: CPU CPU Energy Meter: A tool for energy-aware algorithms engineering. In: Proc. TACAS (2). pp. 126–133. LNCS 12079, Springer (2020). 10.1007/978-3-030-45237-7_8

[18] Bürdek, J., Lochau, M., Bauregger, S., Holzer, A., von Rhein, A., Apel, S., Beyer, D.: Facilitating reuse in multi-goal test-suite generation for software product lines. In: Proc. FASE. pp. 84–99. LNCS 9033, Springer (2015). 10.1007/978-3-662-46675-9_6

[19] Cadar, C., Dunbar, D., Engler, D.R.: Klee: Unassisted and automatic generation of high-coverage tests for complex systems programs. In: Proc. OSDI. pp. 209–224. USENIX Association (2008)

[20] Cadar, C., Nowack, M.: Klee symbolic execution engine in 2019. Int. J. Softw. Tools Technol. Transf. (2020). 10.1007/s10009-020-00570-3

[21] Chalupa, M., Novák, J., Strejček, J.: Symbiotic 8: Parallel and targeted test generation (competition contribution). In: Proc. FASE. LNCS 12649, Springer (2021)

[22] Chalupa, M., Strejček, J., Vitovská, M.: Joint forces for memory safety checking. In: Proc. SPIN. pp. 115–132. Springer (2018). 10.1007/978-3-319-94111-0_7

[23] Chowdhury, A.B., Medicherla, R.K., Venkatesh, R.: VeriFuzz: Program-aware fuzzing (competition contribution). In: Proc. TACAS (3). pp. 244–249. LNCS 11429, Springer (2019). 10.1007/978-3-030-17502-3_22

[24] Cok, D.R., Déharbe, D., Weber, T.: The 2014 SMT competition. JSAT **9**, 207–242 (2016)

[25] Gadelha, M.R., Menezes, R., Cordeiro, L.: Esbmc 6.1: Automated test-case generation using bounded model checking. Int. J. Softw. Tools Technol. Transf. (2020). 10.1007/s10009-020-00571-2

[26] Godefroid, P., Sen, K.: Combining model checking and testing. In: Handbook of Model Checking, pp. 613–649. Springer (2018). 10.1007/978-3-319-10575-8_19

[27] Harman, M., Hu, L., Hierons, R.M., Wegener, J., Sthamer, H., Baresel, A., Roper, M.: Testability transformation. IEEE Trans. Software Eng. **30**(1), 3–16 (2004). 10.1109/TSE.2004.1265732

[28] Holzer, A., Schallhart, C., Tautschnig, M., Veith, H.: How did you specify your test suite. In: Proc. ASE. pp. 407–416. ACM (2010). 10.1145/1858996.1859084

[29] Jaffar, J., Maghareh, R., Godboley, S., Ha, X.L.: TracerX: Dynamic symbolic execution with interpolation (competition contribution). In: Proc. FASE. pp. 530–534. LNCS 12076, Springer (2020). 10.1007/978-3-030-45234-6_28

[30] Jaffar, J., Murali, V., Navas, J.A., Santosa, A.E.: Tracer: A symbolic execution tool for verification. In: Proc. CAV. pp. 758–766. LNCS 7358, Springer (2012). 10.1007/978-3-642-31424-7_61

[31] Jakobs, M.C., Richter, C.: CoVeriTest with adaptive time scheduling (competition contribution). In: Proc. FASE. LNCS 12649, Springer (2021)

[32] Kifetew, F.M., Devroey, X., Rueda, U.: Java unit-testing tool competition: Seventh round. In: Proc. SBST. pp. 15–20. IEEE (2019). 10.1109/SBST.2019.00014

[33] Kim, H.: Fuzzing with stochastic optimization (2020), Bachelor’s Thesis, LMU Munich

[34] King, J.C.: Symbolic execution and program testing. Commun. ACM **19**(7), 385–394 (1976). 10.1145/360248.360252

[35] Le, H.M.: Llvm-based hybrid fuzzing with LibKluzzer (competition contribution). In: Proc. FASE. pp. 535–539. LNCS 12076, Springer (2020). 10.1007/978-3-030-45234-6_29

[36] Lemberger, T.: Plain random test generation with PRTest. Int. J. Softw. Tools Technol. Transf. (2020)

[37] Liu, D., Ernst, G., Murray, T., Rubinstein, B.: Legion: Best-first concolic testing (competition contribution). In: Proc. FASE. pp. 545–549. LNCS 12076, Springer (2020). 10.1007/978-3-030-45234-6_31

[38] Ruland, S., Lochau, M., Jakobs, M.C.: HybridTiger: Hybrid model checking and domination-based partitioning for efficient multi-goal test-suite generation (competition contribution). In: Proc. FASE. pp. 520–524. LNCS 12076, Springer (2020). 10.1007/978-3-030-45234-6_26

[39] Song, J., Alves-Foss, J.: The DARPA cyber grand challenge: A competitor’s perspective, part 2. IEEE Security and Privacy **14**(1), 76–81 (2016). 10.1109/MSP.2016.14

[40] Stump, A., Sutcliffe, G., Tinelli, C.: StarExec: A cross-community infrastructure for logic solving. In: Proc. IJCAR, pp. 367–373. LNCS 8562, Springer (2014). 10.1007/978-3-319-08587-6_28

[41] Sutcliffe, G.: The CADE ATP system competition: CASC. AI Magazine **37**(2), 99–101 (2016)

[42] Visser, W., Păsăreanu, C.S., Khurshid, S.: Test-input generation with Java PathFinder. In: Proc. ISSTA. pp. 97–107. ACM (2004). 10.1145/1007512.1007526

[43] Wendler, P., Beyer, D.: sosy-lab/benchexec: Release 3.6. Zenodo (2021). 10.5281/zenodo.4317433

